# Differential and Combined Therapeutic Effects of Mesenchymal Stem Cells and Glutathione on Methotrexate‐Induced Mucositis

**DOI:** 10.1002/jbt.71023

**Published:** 2026-07-12

**Authors:** Halime Tozak Yıldız, Hilal Akalın, Zeynep Burçin Gönen, Özge Göktepe, Eda Köseoğlu, Nur Seda Gökdemir, Arzu Yay, Munis Dündar

**Affiliations:** ^1^ Institute of Health Sciences, Stem Cell Sciences Doctoral Program Erciyes University Kayseri Turkey; ^2^ Faculty of Medicine, Department of Histology and Embryology Kirsehir Ahi Evran University Kırsehir Turkey; ^3^ Faculty of Medicine, Department of Medical Genetics Erciyes University Kayseri Turkey; ^4^ Department of Oral and Maxillofacial Surgery, Faculty of Dentistry Erciyes University Kayseri Turkey; ^5^ Faculty of Medicine, Department of Histology and Embryology Erciyes University Kayseri Turkey; ^6^ Genome and Stem Cell Center Erciyes University Kayseri Turkey

**Keywords:** antioxidant response, glutathione, inflammation, mesenchymal stem cells, methotrexate, mucositis

## Abstract

Methotrexate (MTX) is commonly used in chemotherapy but induces severe side effects, such as intestinal mucositis, which is characterized by oxidative stress, inflammation, and epithelial cell death. Mesenchymal stem cells (MSCs) and glutathione (GSH) are potential therapeutic agents that could alleviate these effects. In this study, 30 adult female Wistar‐Albino rats were assigned to five groups (*n* = 6) and received either an intraperitoneal dose of MTX (45 mg/kg) followed by treatments of MSCs, GSH, or a combination. MSCs were isolated and confirmed for multipotent differentiation capabilities. The effects of the treatments were assessed through histopathological analysis, immunohistochemistry, biochemical analyses, and gene expression using RT‐qPCR. The MTX group showed significant mucosal damage, including villus atrophy, epithelial shedding, and increased fibrosis. However, treatment groups demonstrated partial preservation of villus architecture and reduced histopathological damage compared with the MTX group. MTX administration also markedly increased inflammatory and apoptotic markers, including TNF‐α, IL‐1β, and caspase‐3, whereas these elevations were reduced to varying extents following MSC and/or GSH treatment. Additionally, MTX elevated malondialdehyde (MDA) levels and decreased antioxidant enzyme activities, MSC and/or GSH treatments attenuated oxidative stress and improved antioxidant parameters. In conclusion, the combination of MSCs and GSH treatment attenuated MTX‐induced intestinal injury through reduction of oxidative stress, inflammation and apoptosis and enhancement of mucosal recovery. The findings suggest that MSC and GSH may exert complementary protective effects against MTX‐induced intestinal toxicity; however, definitive superiority of the combined treatment over monotherapies was not consistently supported by the statistical analyses. However, further studies with larger sample sizes and mechanistic studies are needed to elucidate the underlying interactions and to confirm translational applicability.

## Graphical abstract

Methotrexate (MTX) induces intestinal mucositis through oxidative stress, NF‐κB–mediated inflammation, and epithelial apoptosis. In a rat model, post‐injury administration of mesenchymal stem cells (MSCs) and reduced glutathione (GSH) significantly attenuated mucosal damage. GSH restored redox balance by reducing lipid peroxidation (↓MDA) and enhancing antioxidant enzymes (↑SOD, CAT, GSH‐Px), while MSCs suppressed pro‐inflammatory cytokines (↓TNF‐α, ↓IL‐1β), reduced apoptotic signaling (↓caspase‐3), and promoted tissue repair via paracrine mechanisms. The combined MSC + GSH therapy demonstrated superior preservation of villus architecture and mucosal integrity compared to monotherapy, indicating a synergistic therapeutic effect against MTX‐induced intestinal injury.

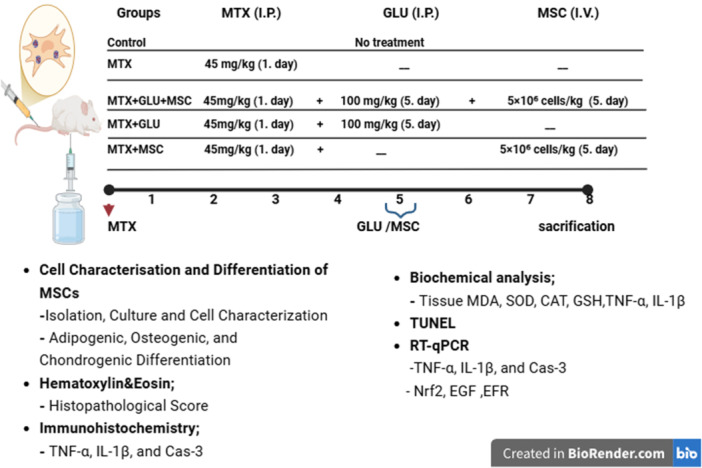

## 1 Introduction

Mucositis is one of the most common and debilitating adverse effects of cancer therapy, particularly chemotherapy and/or radiotherapy, and is characterized by painful inflammation, erythema, and ulceration of the mucosal lining of the gastrointestinal (GI) tract due to mucosal barrier injury [1, 2]. Due to their high mitotic index, the mucosal membranes of the GI system are particularly sensitive to the effects of chemotherapy, which, as a result of the rapid epithelial turnover, leads to inflammation in the epithelial cells of the mucosa [3]. The occurrence of severe mucositis during high‐dose treatments may necessitate interruptions in chemotherapy, thereby adversely affecting prognosis and survival. Moreover, mucositis represents one of the most significant dose‐limiting factors in chemotherapy [4].

GI mucosal epithelium has a high proliferative capacity, with the entire mucosal layer renewing approximately every 4–5 days. The cellular turnover of the GI epithelium is faster than that of the oral mucosa. Consequently, in patients undergoing oncological treatment, GI mucositis typically appears first following chemotherapy. Indirect mechanisms, such as the generation of reactive oxygen species and mediator molecules, also trigger apoptosis. Apoptotic cell death leads to tissue damage, initiating an inflammatory process in the mucosal tissue [5, 6].

Among chemotherapeutic agents, methotrexate (MTX) is one of the most frequently implicated drugs in the development of GI mucosal injury due to its potent antiproliferative effects on rapidly dividing epithelial cells. MTX, a folic acid antagonist, is used as a chemotherapeutic agent in the treatment of certain types of cancer such as leukemia, lymphoma, and breast cancer, and is also widely used in the management of diseases such as dermatomyositis, psoriasis, rheumatoid arthritis, and sarcoidosis [7, 8]. It has been determined that administration of MTX to rats leads to a decrease in glutathione (GSH) levels, which exhibits antioxidant properties, and an increase in myeloperoxidase (MPO) activity, an indicator of inflammatory response, as well as in malondialdehyde (MDA) levels, an indicator of lipid peroxidation [9, 10].

Mesenchymal stem cells (MSC) exhibit anti‐inflammatory, antiapoptotic, antifibrotic, and immunomodulatory properties due to their ability to secrete trophic factors such as VEGF, TGF‐β, and various interleukins [11, 12]. Therefore, they are multipotent stem cells that can be utilized in clinical and preclinical studies of degenerative and immune‐mediated diseases [13, 14, 15]. MSC possess regenerative properties in organ damage induced by low‐dose chemotherapeutic agents under both in vivo and in vitro conditions [16]. In a study, it was demonstrated that in a rat myocardial infarction model, the rate of apoptosis in the peri‐infarct region was significantly reduced following primary transplantation of either MSCs or anoxia‐preconditioned MSCs, as assessed by TUNEL assay [17]. MSC may exert their effects through the paracrine action of secreted growth factors and cytokines that modify the cellular microenvironment, reduce oxidative stress, limit tissue damage, and promote tissue repair [18, 19, 20].

Following MTX‐induced injury, intestinal mucosal repair is regulated by several key molecular pathways controlling inflammation, apoptosis, and epithelial restitution. Central to this process are nuclear factor erythroid 2‐related factor 2 (Nrf2), epidermal growth factor receptor (EFR), and epidermal growth factor (EGF), which govern oxidative stress reduction, cytoprotection, and epithelial proliferation and migration, respectively [21, 22, 23]. However, during chemotherapy‐associated mucositis, these pathways become dysregulated, leading to impaired healing and prolonged inflammation [24]. Therapeutic interventions such as MSC supplementation have demonstrated promising results in restoring the expression of these critical genes, enhancing wound healing and mitigating inflammation [25].

The severe side effects of antineoplastic agents reduce patients' quality of life and may even lead to mortality. Although some treatment‐related toxicities have been relatively controlled, mucositis remains one of the most challenging adverse effects, often leading to dose reductions or treatment interruptions. Various therapeutic agents have been tested for the prevention and management of mucositis; however, their efficacy has generally been limited. Therefore, new strategies with higher efficacy and safety profiles are urgently needed. In this context, the present study aims to evaluate the potential protective effects of MSCs and glutathione administered individually or in combination on MTX‐induced intestinal mucositis, focusing on their antioxidative and regenerative mechanisms.

## 2 Methods

### 2.1 Animals and Experimental Groups

Thirty adult female Wistar–Albino rats (8−10 weeks, 150–210 g) were used in study. Animals were Animals were obtained from an accredited breeding facility and acclimatized prior to the experiment. Rats were housed under controlled environmental conditions (22°C ± 2°C, 12 h light/dark cycle, 55% ± 5% humidity) with ad libitum access to standard chow and water. All experimental procedures were approved by the Erciyes University Local Ethics Committee for Animal Experiments, in Kayseri, Türkiye (Approval No: 21/248) and were conducted in accordance with the ARRIVE guidelines.

The experimental unit was defined as a single animal. Sample size (*n* = 6 per group) was based on previously published studies with similar experimental designs [9, 16], in accordance with the principles of reduction.

Animals were randomly allocated into five experimental groups (*n* = 6 per group) to minimize allocation bias. The study groups were designed as follows:

- Control group: received no pharmacological or cellular intervention.
- MTX group: received a single intraperitoneal (IP) injection of MTX (45 mg/kg) on Day 1 [26, 27].
- MTX + MSC group: received MTX (45 mg/kg, IP) on Day 1 followed by a single intravenous (IV) administration of MSCs (MSCs; 5 × 10^6^ cells/rat) on Day 5 [28, 29].
- MTX + GLU group: received MTX (45 mg/kg, IP) on Day 1 and reduced glutathione (100 mg/kg, IP) on Day 5 [30, 31].
- MTX + MSC + GLU group: received MTX (45 mg/kg, IP) on Day 1, followed by combined treatment with MSCs (5 × 10^6^ cells/rat, IV) and reduced glutathione (100 mg/kg, IP) on Day 5.

All interventions were administered as single‐dose applications on the designated days to avoid repeated handling and chronic injection‐related stress. Animals were monitored throughout the experimental period for general health and welfare status.

All animals were euthanized on Day 8. The interval between the final intervention and tissue collection (3–7 days) was designed to allow recovery from acute procedural effects and to minimize potential confounding effects of transient handling‐ or injection‐induced physiological stress between groups.

Bone marrow‐derived MSCs were produced GENKÖK, Erciyes University, in Türkiye. The study was supported by the Erciyes University Scientific Research Projects Coordination Unit (Project No: TDK‐2022‐11854).

### 2.2 Isolation, Culture and Cell Characterization of Rat Bone Marrow‐Derived MSCs

Bone marrow was harvested from the femurs and tibias of two 8‐week‐old rats, diluted with PBS to 30 mL, and centrifuged at 300*g* for 5 min. The supernatant was filtered through a 70 μm strainer (Falcon, Switzerland). Cells were washed and cultured in Dulbecco's Modified Eagle's medium (DMEM) supplemented with 10% fetal bovine serum (FBS, Gibco, Grand Island, NY), penicillin and streptomycin (100 IU/mL), and 1% glutamine. After reaching 70%–80% confluency, cells were trypsinized and passaged. Passage 3 cells were used for the in vivo experiments. At passage 3, cells were stained with antibodies (BD Biosciences, San Jose, CA, USA) against CD11b, CD73, CD90, and CD105. Monoclonal antibodies were used for the characterization of the MSCs by Flow cytometry (Beckman Coulter, Brea, CA, USA), and the data were analyzed with Kaluza software (Beckman Coulter, USA).

### 2.3 Adipogenic, Osteogenic, and Chondrogenic Differentiation

To assess the multipotent differentiation potential of third‐passage rat bone marrow‐derived MSCs, adipogenic, osteogenic, and chondrogenic protocols were applied. For adipogenesis, cells at 90%–95% confluency were cultured for 21 days with adipogenic induction and maintenance media (Lonza, Basel, Switzerland), and lipid droplets were visualized using the AdipoRed kit (Assay Reagent, Lonza). Osteogenesis was induced by incubating the cells for 10 days in osteogenic medium (Lonza, Basel, Switzerland), with calcium deposits detected using 1% Alizarin Red staining. For chondrogenesis, 5 × 10^5^ cells were pelleted and cultured in chondrogenic medium (Lonza, Basel, Switzerland) for 21 days; cryosections of formed cartilage were stained with Safranin O to confirm proteoglycan formation.

### 2.4 Biochemical Analysis via Enzyme‐Linked Immunosorbent Assay (ELISA)

Intestinal tissue samples stored at –80°C were transferred into 0.01 M phosphate‐buffered saline (PBS, pH 7.4) for homogenization. The homogenates were centrifuged at 134*g* for 10 min at 4°C. The supernatants were used for biochemical assessments according to the manufacturer's protocols of the ELISA kits. Antioxidant enzyme activities in intestinal tissue were evaluated by measuring Glutathione Peroxidase (GSH‐Px; Cat. No: 201‐11‐5104, Sunred Bio, Shanghai, China), Superoxide Dismutase (SOD; Cat. No: 201‐11‐0160, Sunred Bio, Shanghai, China), Catalase (CAT; Cat. No: 201‐11‐5106, Sunred Bio, Shanghai, China), Tumor Necrosis Factor‐alpha (TNF‐α; Cat. No: 201‐11‐0326), and Interleukin‐6 (IL‐6; Cat. No: 201‐11‐0104, Sunred Bio, Shanghai, China). Lipid peroxidation was assessed by quantifying Malondialdehyde (MDA; Cat. No: 201‐11‐0157, Sunred Bio, Shanghai, China) concentrations.

### 2.5 Histological Examination

Intestinal tissues were fixed in 10% neutral formalin for 48 h, dehydrated with a graded ethanol series, and embedded in paraffin. 5 μm sections were cut using a Leica RM2000 microtome (Leica Microsystems, Wetzlar, Germany) and stained with hematoxylin and eosin (H&E) to evaluate general histomorphological features. The tissues were examined under a light microscope using a blinded evaluation protocol. After H&E staining, intestinal mucosal damage was assessed based on standardized scoring criteria. Ten randomly selected areas from five different locations per section were evaluated. Histopathological scoring was performed based on three parameters [1]: degeneration of crypts and surface epithelium [2], structural deterioration and epithelial shedding, and [3] inflammatory cell infiltration in the lamina propria. Each parameter was scored as follows: 0 = none, 1 = mild, 2 = moderate, 3 = severe [32].

### 2.6 Immunohistochemistry

To detect Tumor necrosis factor alpha (TNF‐α), Interleukin‐1 beta (IL‐1β), and Caspase‐3 (Cas‐3) expression in intestinal tissues, the streptavidin‐biotin‐peroxidase method was employed using the Lab Vision UltraVision Detection System (TA‐125‐HL). Paraffin‐embedded intestinal sections (5 µm) were deparaffinized in xylene, rehydrated through a graded ethanol series, rinsed in deionized water and PBS, and treated with 3% hydrogen peroxide to block endogenous peroxidase activity. Antigen retrieval was performed by heating sections in 5% sodium citrate buffer at 600 W for 15 min. Primary antibodies TNF‐α (Cat. No: bs‐6434R; 1:150, Abcam, Cambridge, UK), IL‐1β (Cat. No: bs‐6319R; 1:200, Bioss, Beijing, China), and cleaved Cas‐3 (Cat. No: 87938, 1:500, Cell Signaling Technology, Danvers, MA, USA) were applied overnight at 4°C. The next day, biotinylated secondary antibodies were incubated, followed by visualization using 3,3′‐diaminobenzidine (DAB) substrate. Sections were counterstained with Gill's hematoxylin. Each section was evaluated under a light microscope, and images were captured from five different regions of the intestinal tissue. Immunoreactivity intensities for TNF‐α, IL‐1β, and Cas‐3 were quantified using ImageJ software (NIH, USA).

### 2.7 Intestinal TUNEL Assay

Apoptotic cell detection in intestinal tissue sections was carried out using the TUNEL (Terminal deoxynucleotidyl transferase dUTP nick end labeling) method, which labels DNA strand breaks indicative of apoptosis. The ApopTag Fluorescein In Situ Apoptosis Detection Kit (EMD Millipore, Darmstadt, Germany) was used following the manufacturer's instructions. Paraffin‐embedded sections mounted on poly‐L‐lysine–coated slides were deparaffinized and washed three times with PBS (5 min each). Sections were incubated with the TUNEL reaction mixture for 1 h at 37°C in a dark, humidified chamber. Nuclear counterstaining was performed with 4′,6‐diamidino‐2‐phenylindole (DAPI) to visualize all cell nuclei. Fluorescence imaging was conducted using an Olympus BX51 microscope (Tokyo, Japan). Ten randomly selected high‐power fields (×200) per section were analyzed. The number of TUNEL‐positive cells was quantified using ImageJ software (NIH, Bethesda, MD, USA) [33].

Blinded histopathological evaluation was performed to reduce observer bias.

### 2.8 RT‐qPCR Analysis

Total RNA was extracted from rat intestinal tissues using TRIzol reagent (Thermo Fisher Scientific, Waltham, MA, USA) and purified with the Qiagen RNeasy Mini Kit (Cat. No: 166031400, Qiagen Hilden, Germany). RNA quality and concentration were verified using a NanoDrop spectrophotometer (Thermo Fisher, Scientific, Waltham, MA, USA). cDNA was synthesized using a TransGen Biotech kit (Cat. No: AE301‐03, TransGen Biotech, Beijing, China), and RT‐qPCR was performed on a Roche LightCycler LC480 (Roche Diagnostics, Mannheim, Germany) system to analyze the expression of *TNF‐α, Cas‐3, IL‐1β, Nrf2, EGF, and EFR* (Table 1). Reactions contained cDNA, gene‐specific primers, SYBR Green Master Mix, and RNase‐free water, run in triplicate under the following conditions: 95°C for 10 min (initial denaturation), followed by 40 cycles of 95°C for 15 s, 60°C for 30 s, and 72°C for 30 s. Gene expression was normalized to β‐actin (ACTB), and relative quantification was performed using the 2^−ΔΔCt method [33].

**Table 1:** Quantitative RT‐PCR primer sequences.

Target gene	Nucleotide sequence (5′−3′)
*TNF‐α*	F: TAC TGA ACT TCG GGG TGA TTG GTC C
	R: CAG CCT TGT CCC TTG AAG AGA ACC
*IL‐1 β*	F: GGA ACC CGT GTC TTC CTA AAG
	R: CTG ACT TGG CAG AGG ACA AA
*Cas‐3*	F: AAT TCA AGG GAC GGG TCAT G
	R: GCT TGT GCG CGT ACA GTT TC
*Nrf2*	F: ATTGCTGTCCATCTCTGTCAG
	R: GCTATTTTCCATTCCCGAGTTAC
*EGF*	F: TGA CTA TGA CGG TGG CTC CAT CC
	R: CCC AGT GTG TTT GTC GGC TAT CC
*EFR (EGFR)*	F: TGG AGA GAA TCC CTT TGG AG
	R: TGT TGC TAA ATC GCA CAG C

Abbreviations: Cas‐3, caspase‐3; EFR, epidermal growth factor receptor; EGF, epidermal growth factor; IL‐1β, interleukin‐1 beta; Nrf2, nuclear factor‐erythroid factor 2; TNF‐α, tumor necrosis factor alpha.

Biochemical, histopathological, immunohistochemical, TUNEL and RT‐qPCR analyses were done on intestinal tissues collected from all animals in each experimental group (*n* = 6/group). Technical triplicates were performed for each sample for the RT‐qPCR analyses.

### 2.9 Statistical Analysis of the Data

The results obtained from analyses were evaluated using the GraphPad Prism 9.0 statistical program. The Shapiro−Wilk test was performed to assess the normality of the data distribution. For comparisons involving multiple groups, one‐way analysis of variance (ANOVA) and the Kruskal−Wallis test were utilized. Post hoc analyses were performed using Tukey's multiple comparison test following ANOVA and Dunn's multiple comparison test following the Kruskal–Wallis test. A p‐value of less than 0.05 was considered statistically significant.

## 3 Results

### 3.1 Cell Characterisation and Differentiation of MSCs

**Figure 1:**
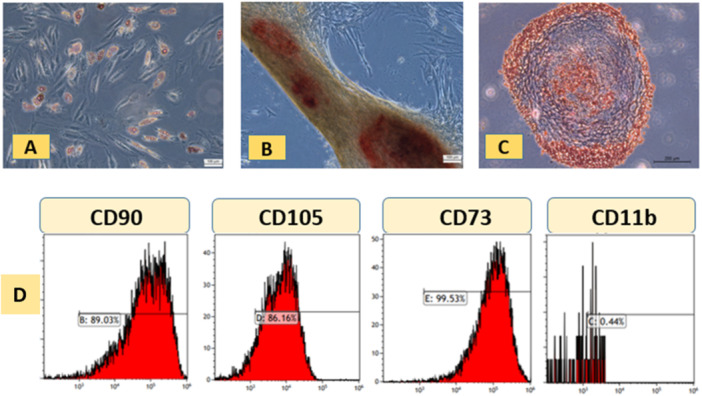
Characterization of MSCs. Morphology and differentiation potential were assessed under a light microscope; (A) Adipogenic differentiation visualized by AdipoRed staining. (B) Osteogenic differentiation visualized by Alizarin Red staining. (C) Chondrogenic differentiation visualized by Safranin O staining. Scale bars: A, B = 100 µm; C = 200 µm. (D) Flow cytometric analysis of surface markers (CD90, CD105, CD73, and CD11b) on bone marrow‐derived mesenchymal stem cells (BMSCs).

The multipotent differentiation potential of MSCs was confirmed by adipogenic, osteogenic, and chondrogenic induction. Intracellular lipid accumulation indicating adipogenesis was demonstrated by AdipoRed staining (Figure 1A), while calcium deposits in osteogenically differentiated cells were visualized using Alizarin Red staining (Figure 1B). Chondrogenic differentiation was evidenced by Safranin O staining, which revealed a proteoglycan‐rich extracellular matrix in cartilage‐like pellets (Figure 1C).

Following flow cytometric characterization, it was determined that CD11b was not expressed (0.44%), whereas CD105 (86.16%), CD73 (99.53%), and CD90 (89.03%) surface markers were strongly expressed (Figure 1D).

### 3.2 Histopathological Changes in Intestinal Architecture

**Figure 2:**
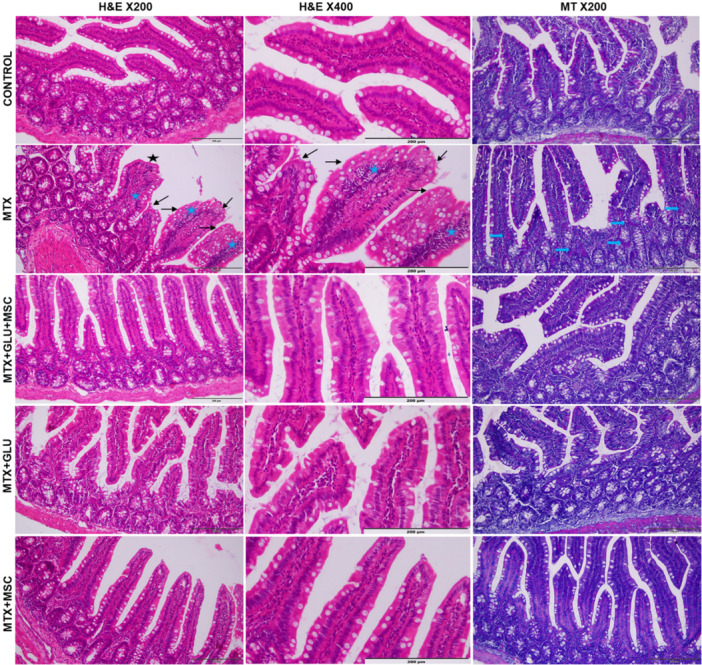
Histological analysis of intestinal sections stained with hematoxylin and eosin (H&E; x200 and x400) and Masson's Trichrome (MT; ×200, scale bar = 200 µm). The control group shows intact mucosa with minimal collagen deposition. The MTX group exhibits severe mucosal damage, including surface epithelial degeneration (black arrow), epithelial shedding (black star), and inflammatory cell infiltration (blue star), accompanied by increased fibrosis (blue arrow). Treatment with MSC and/or GLU largely restored mucosal architecture and reduced collagen deposition. GLU, glutathione; MSC, mesenchymal stem cells; MTX, methotrexate.

In the control group, H&E staining revealed intact villi, preserved epithelial integrity, and regular crypt architecture, while MT staining showed minimal connective tissue and negligible collagen deposition. In contrast, the MTX group exhibited marked villus atrophy, epithelial shedding, crypt degeneration, and mucosal disorganization in H&E sections, accompanied by significant subepithelial collagen accumulation indicative of fibrosis in MT staining. Treatment with MSCs largely preserved villus morphology and reduced inflammation, with MT staining showing decreased collagen content in the MTX + MSC group. Similarly, the MTX + GLU group demonstrated more organized mucosal architecture and reduced fibrotic changes. In the MTX + MSC + GLU group, villus integrity was substantially preserved, inflammatory alterations were markedly reduced, and fibrosis scores were significantly lower compared with the MTX group, although mild residual collagen deposition was still observed relative to controls (Figure 2).

Histopathological examination revealed marked mucosal damage, crypt degeneration, and epithelial loss in the MTX group compared with the control group. In contrast, animals treated with MSC and/or glutamine showed partial preservation of mucosal architecture and a noticeable reduction in inflammatory cell infiltration. Quantitative scoring confirmed that degeneration of crypts and surface epithelium, mucosal structural deterioration, and lamina propria inflammation and fibrosis scores were significantly increased in the MTX group (*p* < 0.001 for all parameters; fibrosis *p* < 0.01). All treatment groups demonstrated significantly decreased scores compared to MTX alone. Although the MTX + MSC + GLU group exhibited the lowest fibrosis scores and generally improved histological appearance, differences among the treatment groups were not statistically significant (Table 2).

**Table 2:** Histopathological scoring of intestinal damage.

Group	Degeneration of crypts and surface epithelium	Structural deterioration of the mucosa and epithelial shedding	Inflammatory cell infiltration in the lamina propria	Fibrosis score (MT staining)
CONTROL	0.17 ± 0.37^a^	0.17 ± 0.37^a^	0.00 ± 0.00^a^	0.17 ± 0.49^a^
MTX	2.83 ± 0.37^c^	3.00 ± 0.00^c^	2.67 ± 0.37^c^	2.50 ± 0.68^c^
MTX + MSC + GLU	1.33 ± 0.47^b^	1.33 ± 0.47^b^	1.50 ± 0.50^b^	1.17 ± 0.49^b^
MTX + GLU	1.67 ± 0.47^b^	1.50 ± 0.50^b^	1.67 ± 0.47^b^	1.33 ± 0.64^b^
MTX + MSC	1.50 ± 0.50^b^	1.50 ± 0.50^b^	1.50 ± 0.50^b^	1.50 ± 0.68^b^
*p* value	< 0.001	< 0.001	< 0.001	< 0.01

*Note:* Values are expressed as mean ± standard deviation (SD). Groups sharing the same superscript letter within the same column are not significantly different, whereas groups with different superscript letters differ significantly (*p* < 0.05). Statistical analysis was performed using one‐way ANOVA followed by Tukey's multiple comparison test.

Abbreviations: GLU, glutathione; MSC, mesenchymal stem cells; MTX, methotrexate.

### 3.3 Immunohistochemical Modulation of Inflammatory and Apoptotic Markers

**Figure 3:**
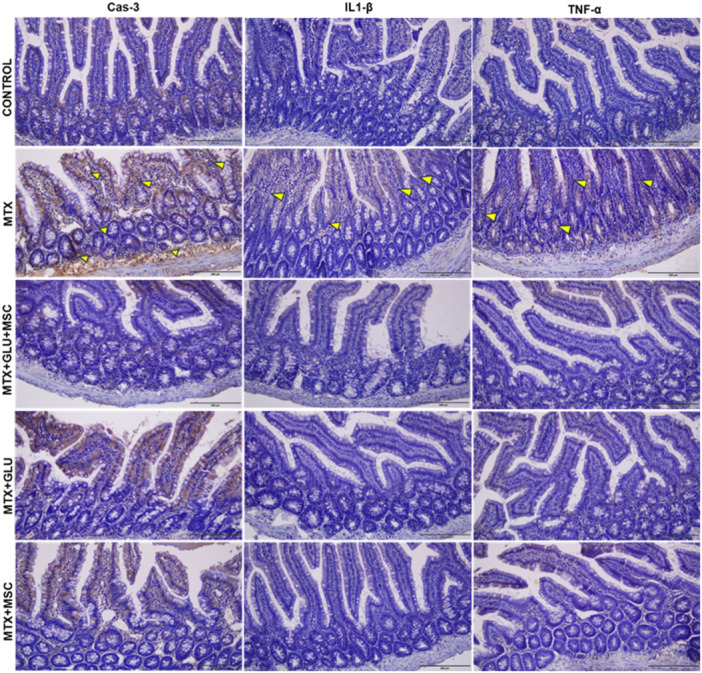
Immunohistochemical analysis of intestinal sections. (Olympus BX51, Tokyo, Japan. X200: Scale bar; 200 µm) IHC staining for cleaved Cas‐3, IL‐1β, and TNF‐α, in jejunal tissue. Arrowheads indicate positive inflammatory cells in tissue. Note strong epithelial and lamina propria staining in the MTX group and reduced immunoreactivity in MSC, GLU, and MSC + GLU treatment groups. Avidin‐biotin peroxidase technique. Cas‐3, caspase‐3; GLU, glutathione; IL‐1β, Interleukin‐1 beta; MSC, mesenchymal stem cells; MTX, methotrexate; TNF‐α, tumor necrosis factor alpha.

In the control group, TNF‐α, IL‐1β, and Cas‐3 immunoreactivities in the jejunal mucosa were low, with weak staining mainly observed in the villus epithelium and limited presence in the lamina propria. In the MTX‐treated group, all markers showed a marked increase. TNF‐α and IL‐1β expression was predominantly localized in the cytoplasm of epithelial cells at the villus tips and in lamina propria cells, while Cas‐3 positivity, indicative of apoptosis, was observed in epithelial cells at the villus tips and within the crypt epithelium. Immunoreactivity was reduced to different extents in the MSC, GLU, and combination treatment groups. The MTX + MSC + GLU group showed lower staining intensities of TNF‐α and IL‐1β than the MTX group, and Cas‐3 immunoreactivity was also trending downwards. Partial decreases in marker expression were also observed in the MTX + GLU and MTX + MSC groups (Figure 3).

**Figure 4:**
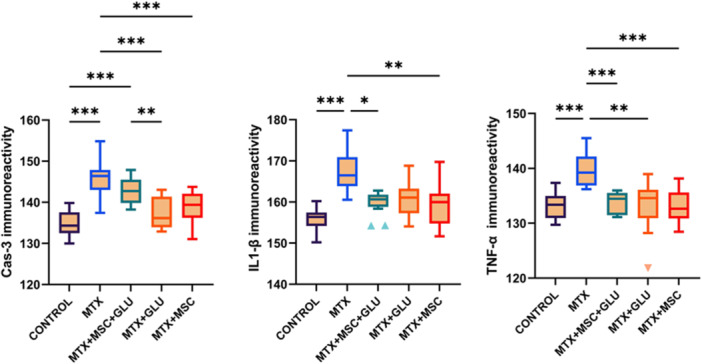
The immunoreactivity scores for TNF‐α, IL‐1β, and Cas‐3 in intestinal tissues. MTX administration significantly increased TNF‐α, IL‐1β, and Cas‐3 immunoreactivity compared with the control group. (*p* < 0.001). Treatment with MSC and/or GLU reduced immunoreactivity levels to varying extents depending on the marker analyzed. Data are presented as mean ± SD. Statistical analysis was performed using one‐way ANOVA followed by Tukey's multiple comparison test. *p < 0.05, **p < 0.01, ***p < 0.001. Cas‐3, caspase‐3; GLU, glutathione; IL‐1β, interleukin‐1 beta; MSC, mesenchymal stem cells; MTX, methotrexate; TNF‐α, tumor necrosis factor alpha.

Quantitative analysis demonstrated that Cas‐3, IL‐1β, and TNF‐α immunoreactivity scores were significantly increased in the MTX group compared with the control group (*p* < 0.001). TNF‐α immunoreactivity was significantly down‐regulated in all the treatment groups (MTX + MSC + GLU and MTX + MSC groups (*p* < 0.001); MTX + GLU group (*p* < 0.01)) as compared to MTX. IL‐1β immunoreactivity was markedly reduced in the MTX + MSC + GLU (*p* < 0.05) and MTX + MSC groups (*p* < 0.01), but only slightly in the MTX + GLU group. Large reductions were observed for Cas‐3 in MTX + GLU and MTX + MSC groups (*p* < 0.001), while the reduction in MTX + MSC + GLU group was not statistically significant. While combination treatment was generally associated with improved histological and immunohistochemical findings, variation in differences between treatment groups was observed depending on the marker analysed (Figure 4).

### 3.4 Changes in Antioxidant Enzymes and Proinflammatory Cytokines

MTX administration induced marked oxidative stress and inflammation in intestinal tissue. MDA levels were significantly elevated in the MTX group compared with the control (*p* < 0.001), whereas SOD, CAT, and GSH‐Px levels were significantly reduced (all *p* < 0.001). There was a significant decrease in the MDA levels in the MTX + MSC + GLU and MTX + MSC groups (*p* < 0.001), and the restoration of antioxidant parameters was different among the treatment regimens. SOD activity was significantly higher in the MTX + MSC group (*p* < 0.001). CAT activity was significantly increased in the MTX + MSC + GLU, MTX + MSC, and MTX + GLU groups (*p* < 0.01). GSH‐Px levels were markedly increased in all three treatment groups (*p* < 0.01).

**Figure 5:**
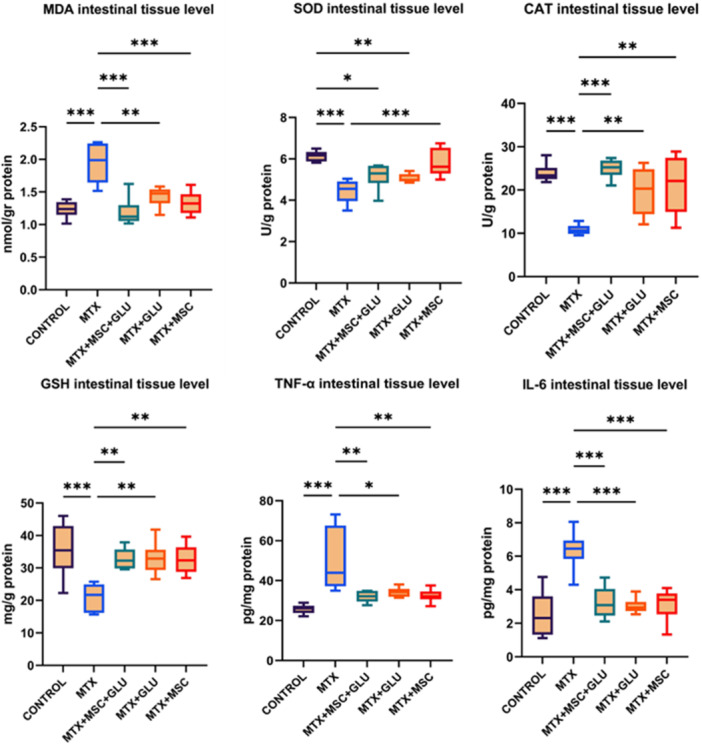
Intestinal tissue levels of oxidative stress (MDA), antioxidant enzymes (SOD, CAT, GSH‐Px), and proinflammatory cytokines (TNF‐α, IL‐6) following MTX administration and subsequent treatments with MSC, GLU, or their combination. Data are presented as mean ± SD. Statistical analysis was performed using one‐way ANOVA followed by Tukey's multiple comparison test. *p < 0.05, **p < 0.01, ***p < 0.001. CAT, catalase; GLU, glutathione; GSH, glutathione peroxidase; IL‐6, interleukin‐6; MDA, malondialdehyde; MSC, mesenchymal stem cells; MTX, methotrexate; SOD, superoxide dismutase; TNF‐α, tumor necrosis factor alpha.

Regarding inflammatory cytokines, the MTX group exhibited a significant increase in TNF‐α and IL‐6 levels (*p* < 0.001). Such decreases in these cytokine levels were observed in all treatment groups compared to the MTX group. Although the MTX + MSC + GLU group showed lower TNF‐α and IL‐6 values and an improved overall biochemical profile, no statistically significant differences were observed between the treatment groups (Figure 5).

### 3.5 TUNEL Assay Findings and Apoptotic Cell Analysis

**Figure 6:**
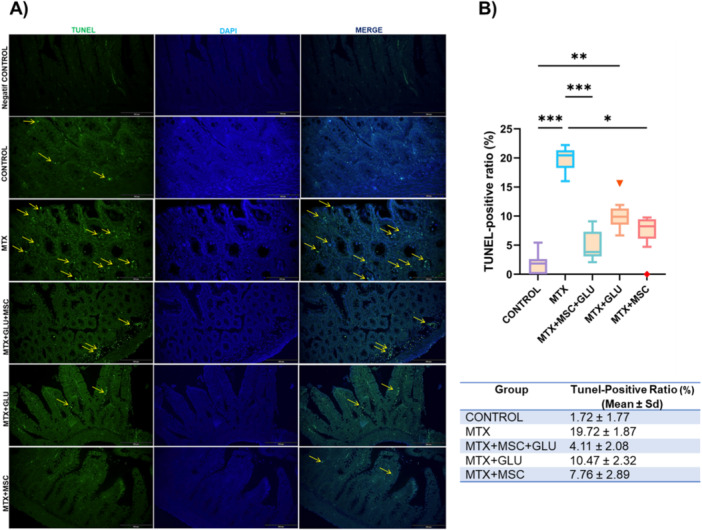
(A) Representative TUNEL staining of jejunal tissue sections showing apoptotic nuclei (green) and DAPI‐stained nuclei (blue) at ×200 magnification (scale bar = 100 µm). The MTX group exhibits intense TUNEL positivity in the villus epithelium and lamina propria (yellow arrows), indicating increased apoptosis. In contrast, treatment with MSC, GLU, or their combination markedly reduced TUNEL positivity. Compared with the MTX group. Representative images from the treatment groups demonstrate decreased apoptotic signaling and improved preservation of mucosal architecture relative to MTX‐treated animals. (B) Quantitative analysis of TUNEL‐positive apoptotic cells in intestinal tissue. Data are presented as mean ± SD. Statistical analysis was performed using one‐way ANOVA followed by Tukey's multiple comparison test. *p < 0.05, **p < 0.01, *p < 0.001. GLU, glutathione; MSC, mesenchymal stem cells; MTX, methotrexate.

TUNEL staining revealed distinct differences in apoptotic activity among the groups. In the control group, TUNEL‐positive cells were rarely observed, and the intestinal mucosa exhibited normal epithelial architecture with minimal apoptotic nuclei. In contrast, the MTX group showed a marked increase in TUNEL‐positive nuclei, predominantly localized in the villus tips and lamina propria, indicating extensive DNA fragmentation and apoptotic cell death. Treatment with either MSC or GLU markedly reduced the number of TUNEL‐positive cells compared to the MTX group, although scattered apoptotic nuclei were still evident. The MTX + MSC + GLU treatment group also showed reduced TUNEL reactivity, together with preserved villus morphology and a lower apoptotic signal relative to the MTX group. However, no statistically significant differences were observed among the treatment groups. These findings suggest that MSC and/or GLU treatment attenuates MTX‐induced apoptotic damage in intestinal tissue (Figure 6A).

According to the TUNEL analysis, the proportion of apoptotic cells increased markedly in the MTX‐treated group. In contrast, the MTX + MSC + GLU group showed a significantly reduced level of apoptosis, representing the treatment group that most closely approached the control values (*p* < 0.001). Significant reductions in apoptotic cells were also observed in the MTX + MSC (*p* < 0.05) and MTX + GLU groups compared with the MTX group. When the graph and table are evaluated together, it is evident that the combination therapy (MSC + GLU) exerts the strongest protective effect in reducing MTX‐induced apoptosis (Figure 6B).

### 3.6 Gene Expression Alterations Assessed by qPCR

In the MTX group, *Cas‐3, IL‐1β*, and *TNF‐α* expression levels tended to increase compared with the control group, although not all changes reached statistical significance. *Cas‐3* expression increased 2.07‐fold (*p* = 0.067), while *IL‐1β* and *TNF‐α* showed borderline/significant elevations (1.37‐fold, *p* = 0.05; 1.44‐fold, *p* = 0.02, respectively).

In the MTX + MSC + GLU group, *Cas‐3* expression was reduced to near‐control levels (0.90‐fold, *p* = 0.02). *IL‐1β* expression also approached baseline levels (1.08‐fold, *p* = 0.04), whereas *TNF‐α* showed a mild downward trend (1.17‐fold, *p* = 0.05). In the MTX + MSC group, *Cas‐3* expression decreased significantly (1.00‐fold, *p* = 0.045), while *TNF‐α* demonstrated a non‐significant downward trend (0.60‐fold, *p* = 0.09). In the MTX + GLU group, *TNF‐α* expression was markedly suppressed (0.23‐fold, *p* = 0.011), whereas *Cas‐3* showed a tendency toward reduction without statistical significance (1.15‐fold, *p* = 0.058) (Table 3).

**Table 3:** Real‐time PCR analysis of gene expression across experimental groups.

Genes	MTX	MTX + MSC + GLU	MTX + MSC	MTX + GLU
***Cas‐3***	↑ 2.07 (*p* = 0.067)	↑ 0.90 **(*p*** = **0.02)**	↑ 1.00 **(*p*** = **0.045)**	↑ 1.15 (*p* = 0.058)
***IL‐1β***	↑ 1.37 **(*p*** = **0.05)**	≈ 1.08 **(*p*** = **0.04)**	↑ 1.39 (*p* = 0.51)	↑ 0.87 (*p* = 0.54)
***TNF‐α***	↑ 1.44 **(*p*** = **0.02)**	↓ 1.17 **(*p*** = **0.05)**	↓ 0.60 (*p* = 0.09)	↓ 0.23 **(*p*** = **0.011)**
***Nrf2***	↓ 0.35 (*p* = 0.07)	↑ 0.80 **(*p*** = **0.045)**	↓ 0.52 (*p* = 0.11)	↓ 0.27 **(*p*** = **0.048)**
***EFR***	↓ 0.41 **(*p*** = **0.049)**	↑ 1.80 **(*p*** = **0.03)**	↑ 0.62 (*p* = 0.12)	≈ 1.04 (*p* = 0.80)
***EGF***	↓ 0.43 (*p* = 0.12)	↑ 2.41 **(*p*** = **0.04)**	↑ 0.70 (*p* = 0.18)	↑ 0.82 (*p* = 0.48)

*Note:* Fold‐change values (relative to control) and corresponding *p* values for target gene expression levels measured by real‐time PCR. Upregulation and downregulation patterns are shown for each experimental group in comparison with the control group. Only *p* values < 0.05 were considered statistically significant. Values with *p* > 0.05 were considered trends and reported descriptively.

Abbreviations: Cas‐3, caspase‐3; EFR, epidermal growth factor receptor; EGF, epidermal growth factor; GLU, glutathione; IL‐1β, interleukin‐1 beta; MSC, mesenchymal stem cells; MTX, methotrexate; Nrf2, nuclear factor‐erythroid factor 2; TNF‐α, tumor necrosis factor alpha.

## 4 Discussion

MTX‐induced intestinal toxicity is a multifactorial condition involving oxidative stress, inflammation, and apoptosis. In the present study, MSC and/or GLU treatment attenuated MTX‐induced intestinal injury across histopathological, biochemical, immunohistochemical, and molecular parameters. These findings support the involvement of oxidative stress‐driven inflammatory and apoptotic pathways in MTX toxicity and suggest that both treatments may confer protection through complementary biological mechanisms.

The histopathological alterations observed in the MTX group, including villus atrophy, epithelial desquamation, crypt injury, and increased subepithelial collagen deposition, are consistent with MTX‐induced mucositis and fibrosis mediated by ROS accumulation, epithelial stem‐cell loss, and TGF‐β–driven fibroblast activation [5, 9, 32, 34]. The histological improvement observed after treatment with MSC and/or GLU is consistent with the reported antioxidant, anti‐inflammatory and tissue‐protective properties of these agents. Some histopathological parameters numerically showed more improvement in MSC + GLU group, but statistically significant differences between treatment groups were not demonstrated. MSCs have been shown to promote epithelial regeneration and reduce inflammatory injury [17, 18, 19, 29, 35], and glutathione‐based strategies have been shown to maintain mucosal integrity via redox regulation [31]. These findings collectively suggest that MSC and GLU treatment have the potential to reduce the MTX‐induced intestinal injury and fibrotic changes.

Our immunohistochemical and molecular analyses provide a comprehensive picture of the inflammatory and apoptotic cascade triggered by MTX. We observed a marked upregulation of TNF‐α, IL‐1β, and the executioner protein Cas‐3, a profile consistent with the activation of NF‐κB and MAPK‐dependent pathways which propagate epithelial injury and cell death [36, 37]. This apoptotic response is further amplified beyond direct DNA damage through secondary ROS‐mediated mitochondrial permeability transition, leading to caspase‐dependent DNA fragmentation [38, 39]. Treatment was associated with attenuation of inflammatory and apoptotic markers, although the magnitude and statistical significance of these effects varied by marker analysed. MSCs are known to modulate NF‐κB‐mediated inflammatory responses through paracrine signalling and may reduce mitochondrial apoptotic signalling, while GLU contributes to the maintenance of cellular redox balance and limits oxidative stress‐induced tissue injury [40, 41]. Furthermore, GLU participates in the synthesis of glutathione, thus further contributing to the stabilisation of the intracellular redox environment [41, 42]. These data, taken together, suggest that MSC and GLU treatments may be able to protect against MTX‐induced epithelial injury via partially complementary anti‐inflammatory and antioxidant mechanisms.

The MTX + MSC + GLU group had the lowest TUNEL score, showing a significant decrease of DNA fragmentation and epithelial cell death. While Cas‐3 immunoreactivity in the MTX + MSC + GLU group was not statistically significant compared to the MTX group, this result does not necessarily contradict the TUNEL findings. Cas‐3 is a marker of apoptotic pathway activation, whereas TUNEL staining reflects late‐stage DNA fragmentation during terminal apoptosis. Therefore, a reduction in terminal apoptotic cell death may still coexist with residual Cas‐3 activity. Taken together, these findings suggest that MSC + GLU treatment may contribute more prominently to limiting progression toward terminal apoptosis and tissue destruction rather than completely suppressing early apoptotic signalling.

The marked oxidative stress induced by MTX, characterized by increased lipid peroxidation and impaired antioxidant defenses, is consistent with previous reports describing oxidative injury as a central mechanism of MTX‐induced intestinal toxicity [9, 39, 43]. The treatment with MSC and/or GLU improved the oxidative stress and inflammatory parameters, supporting their protective roles against the intestinal injury induced by MTX. These effects could be associated with the capacity of GLU to promote antioxidant defence mechanisms and redox balance, while MSCs could improve endogenous antioxidant responses and modulate inflammatory pathways in paracrine and immunoregulatory mechanisms. Although some oxidative stress and cytokine parameters improved numerically more in the combined treatment group, statistically significant differences between treatment groups were not consistently observed.

At the transcriptional level, MTX induced a pathological signature characterized by upregulation of inflammatory (*TNF‐α, IL‐1β*) and apoptotic (*Cas‐3*) genes alongside suppression of the antioxidant regulator *Nrf2* and growth‐related genes *EFR* and *EGF* [5, 7, 9, 36, 44, 45]. This dual suppression of cytoprotective and regenerative pathways illustrates the core mechanism of MTX‐induced damage. The molecular changes were modulated to different degrees by therapeutic interventions. Significant restoration of *Nrf2, EFR,* and *EGF* expression levels was associated with the combined MSC + GLU treatment. The reductions in inflammatory and apoptotic gene expression were dependent on the analysed marker. Especially, the expression of *TNF‐α* in MTX + GLU group was remarkably decreased. But the changes of *Cas‐3* and *IL‐1β* in some treatment groups were not always statistically significant. These findings may reflect the complementary biological properties of MSCs and GLU. GLU contributes to glutathione synthesis and maintenance of intracellular redox balance [46, 47], whereas MSCs are known to modulate inflammatory signaling pathways and support tissue repair through paracrine mediators and growth factor‐related mechanisms [16, 19, 48, 49]. Although some molecular parameters numerically improved more significantly in the combined treatment group, the present data do not consistently show a statistically significant superiority over monotherapies for all analysed genes. Thus, the results suggest that MSC and GLU might have a partially cooperative protective role in MTX‐induced intestinal injury at the transcriptional level.

In conclusion, this study demonstrates that post‐injury administration of MSCs and exogenous Glutathione (GLU) provides protective effects against MTX‐induced intestinal damage. Despite being delivered after the onset of mucosal injury, both agents significantly alleviated tissue destruction. Combined MSC + GLU treatment was associated with improvements in several histopathological, biochemical, immunohistochemical, and molecular parameters; however, statistically significant superiority over monotherapies was not consistently demonstrated across all analyses. The therapeutic effects observed with MSC and GLU treatment may be related to their complementary biological activities. Exogenous GLU replenishes intracellular antioxidant capacity and neutralizes accumulated reactive oxygen species, thereby reducing oxidative stress. In parallel, MSCs may modulate inflammatory signaling, attenuate apoptosis‐related pathways, and support mucosal healing through paracrine‐mediated regenerative mechanisms. Accordingly, the combined treatment may contribute to limiting MTX‐induced tissue injury and supporting preservation of mucosal architecture. These findings highlight MSC and GLU treatment as a potentially beneficial therapeutic approach for mitigating MTX‐induced GI toxicity.

However, several limitations should be noted. This study employed a single‐dose treatment design, which does not fully reflect the complexity of clinical mucositis management. Additionally, long‐term outcomes, pharmacokinetic interactions, and potential systemic effects of the combined therapy were not investigated. Future studies incorporating repeated dosing, functional assessments, and mechanistic analyses at the molecular level will be essential to confirm the translational potential of this therapeutic approach.

## Author Contributions

**Halime Tozak Yıldız:** methodology, writing – original draft, data curation, formal analysis, writing – review and editing. **Hilal Akalın:** methodology, writing – review and editing, data curation. **Zeynep Burçin Gönen:** methodology, validation. **Özge Göktepe:** visualization, validation, methodology. **Eda Köseoğlu:** methodology, validation. **Nur Seda Gökdemir:** methodology, validation. **Arzu Yay:** Visualization, validation, data curation. **Munis Dündar:** supervision, data curation, writing – review and editing, funding acquisition, project administration.

## Conflicts of Interest

The authors declare no conflicts of interest.

## Supporting information


Supporting File

## Data Availability

The data sets used and/or analyzed during the current study are available from the corresponding author on reasonable request.
